# Peripheral whole blood lncRNA expression analysis in patients with eosinophilic asthma

**DOI:** 10.1097/MD.0000000000009817

**Published:** 2018-02-23

**Authors:** Yu-Jin Zhu, Dan Mao, Wei Gao, Hong Hu

**Affiliations:** a Respiratory Department, Chinese PLA General Hospital, FuXing Road, Haidian District, Beijing, China; b Tianjin Municipal Corps Hospital of CAPF, WeiGuo, DongLi, Tianjin, China.

**Keywords:** biomarker, eosinophilic asthma, IgE, long noncoding RNA, peripheral whole blood, RNA sequencing

## Abstract

Long noncoding RNA (lncRNA) plays roles in many diseases including asthma. Several lncRNAs function in the early differentiation of T-helper cells. lncRNA controls gene transcription, protein expression, and epigenetic regulation. Of the 4 asthma phenotypes, eosinophilic asthma (EA) is the most common. However, the lncRNAs associated with eosinophilic asthma have yet to be identified.

We designed a study to identify the circulating lncRNA signature in EA samples. We tested whether significant differences in lncRNA expression were observed between blood samples from patients with EA and healthy individuals (control). Gene ontology (GO) and Kyoto Encyclopedia of Genes and Genomes (KEGG) pathway analyses were performed for the lncRNA–mRNA (messenger RNA) co-expression network. lncRNA expression was measured using quantitative real-time PCR (polymerase chain reaction).

A total of 41 dysregulated lncRNAs and 762 dysregulated mRNAs (difference ≥ 2-fold) were found in EA compared to control samples. GO terms and KEGG pathway annotation data revealed that several lncRNAs are significantly associated with EA. KEGG pathway annotation indicated that the pathways most enriched in EA were measles, T cell receptor signaling pathway, peroxisome proliferator activated-receptors (PPAR) signaling pathway, Fc gamma R-mediated phagocytosis, NF (nuclear factor) kappa B signaling pathway, chemokine signaling pathway, and primary immunodeficiency. Using qRT-PCR, lncRNA was confirmed to differ significantly between EA and control samples.

The results presented here show that several lncRNAs may take part in the immune regulation of EA. Whether these lncRNAs can be used as biomarkers needs further study.

## Abstract 2

Supplemental Digital Content is available in the text

## 1 Introduction

### 1.1 Background

Airway inflammation in asthma can be categorized into 4 inflammatory subtypes based on sputum eosinophil and neutrophil proportions. The subtypes are eosinophilic asthma (EA), neutrophilic asthma, mixed granulocytic asthma, and paucigranulocytic asthma.^[1]^ Asthma can also be categorized based on clinical symptoms and eosinophilic degree.^[2]^ The CD4 T-helper cell type 2 (Th2)-mediated pathway orchestrated by the airway epithelium has been recognized as a driving force in allergic EA.^[3,4]^ However, EA can also be underlain by a non-Th2 mechanism involving innate lymphoid cell type 2 (ILC2).^[5,6]^ Both pathways are associated with expression of IgE. Severe asthma is defined as partly or totally unresponsive to asthma treatments, and is always accompanied by an increase in eosinophil granulocytes.^[7]^ The inflammatory mechanisms underlying severe asthma involve multiple cellular compartments with a diversity of disease-driving mechanisms. The disease driver(s) associated with EA remain largely unclear, especially with respect to lncRNAs.^[8,9]^

Genomic analysis has shown that 75% of the human genome is transcribed into RNA, and only 1% of which encode proteins, indicating that a large portion of the genome is dedicated to regulation.^[10,11]^ Among these newly discovered RNA elements, lncRNAs have been identified to have functional roles in a diverse range of cellular functions such as development, differentiation, cell fate, as well as disease pathogenesis.^[12]^ Many lncRNAs have been identified, ranging from 0.2 to 100 kilobases (kb) in length. lncRNA regulates gene transcription and protein expression both genetically and epigenetically, and altered expression results in many diseases. lncRNAs have been shown to be differentially expressed in T cell development and differentiation.^[13]^ Moreover, lncRNAs function in regulating differentiation of DCs and Treg cells,^[14,15]^ which participate in CD4+ T-cell development and activation.^[16]^

### 1.2 Objective

We hypothesized that lncRNA might also be involved in eosinophilic inflammation, and wanted to investigate whether lncRNAs could be developed as prognostic markers in EA. We performed clustering analysis of differentially expressed genes (DEGs) in EA versus control samples to identify driving mechanisms that indicate the significance of the eosinophilic inflammatory profile.

## 2 Methods

### 2.1 Participants

Patients with eosinophilic asthma (EA, n = 9) were selected for inclusion in the study according to the accepted standard (induced sputum eosinophil count >3% and neutrophils <63%).^[1]^ Exclusion criteria included recent (within the past month) respiratory tract infection, recent asthma exacerbation, recent unstable asthma, changes in maintenance therapy, and current smoking (or a history of smoking, within 6 months of cessation). All patients were selected from the People's Liberation Army General Hospital. EA samples were subdivided into a high-expression IgE group (EAH n = 6) and a low-expression IgE group (EAL n = 3). Healthy individuals were selected as control samples (n = 3). Clinical data for individual samples are provided in Table 1. This study was approved by the Ethics Committee of the People's Liberation Army General Hospital. Informed consent was obtained from each donor.

**Table 1:**
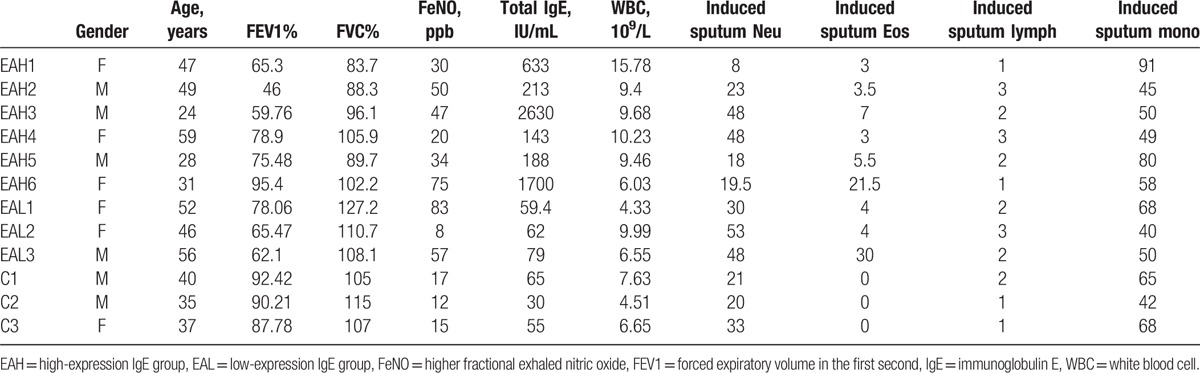
The main clinical and laboratory features of the EA and control samples.

### 2.2 Sputum induction and analysis

Sputum induction was performed with hypertonic saline (4.5%). A fixed sputum induction time of 15 minutes was used for all participants. For inflammatory cell counts, selected sputum (sputum portion separated from saliva) was dispersed using dithiothreitol. The suspension was filtered and a total cell count of leucocytes and cell viability was performed.

### 2.3 RNA isolation, library preparation, and sequencing

Each total cellular RNA was isolated from 4 mL peripheral whole blood samples using 12 mL TRIzol reagent (Invitrogen, Carlsbad, CA) and stored at −80°C until use. RNA degradation and contamination were monitored on 1% agarose gels. RNA purity was checked using the NanoPhotometer spectrophotometer (IMPLEN, CA). RNA concentration was measured using a Qubit RNA Assay Kit in a Qubit 2.0 Fluorometer (Life Technologies, CA). RNA integrity was assessed using the RNA Nano 6000 Assay Kit of the Bioanalyzer 2100 system (Agilent Technologies, CA).

### 2.4 Library preparation for lncRNA sequencing

A total of 3 μg of RNA per sample was used as input material for the RNA sample preparations. Firstly, ribosomal RNA was isolated using the Epicentre Ribo-zero rRNA Removal Kit (Epicentre), and the rRNA-free material was cleaned by ethanol precipitation. Subsequently, sequencing libraries were generated using the rRNA-depleted RNA using a NEBNext Ultra Directional RNA Library Prep Kit for Illumina (NEB) following the manufacturer's recommendations. Briefly, fragmentation was carried out using divalent cations under elevated temperature in NEBNext First Strand Synthesis Reaction Buffer (5X). First strand cDNA was synthesized using a random hexamer primer and M-MuLV Reverse Transcriptase (RNaseH-). Second strand cDNA synthesis was subsequently performed using DNA polymerase I and RNase H. In the reaction buffer, dTTPs were replaced by dUTP. Remaining overhangs were converted into blunt ends via exonuclease/polymerase activities. After adenylation of the 3′ ends of the DNA fragments, a NEBNext Adaptor with a hairpin loop structure was ligated in preparation for hybridization. In order to select cDNA fragments of 150 to 200 bp in length, the library fragments were purified with the AMPure XP system (Beckman Coulter, Beverly). Then 3 μL of USER Enzyme (NEB) was used with size-selected, adaptor-ligated cDNA at 37°C for 15 minutes followed by 5 minutes at 95°C before PCR. PCR was performed with Phusion High-Fidelity DNA polymerase, Universal PCR primers, and Index (X) Primer. At last, products were purified (AMPure XP system) and library quality was assessed on the Agilent Bioanalyzer 2100 system.

### 2.5 Clustering and sequencing

The clustering of the index-coded samples was performed on a cBot Cluster Generation System using a TruSeq PE Cluster Kit v3-cBot-HS (Illumina) according to the manufacturer's instructions. After cluster generation, the libraries were sequenced on an Illumina Hiseq 2500 platform and 125-bp paired-end reads were generated.

### 2.6 Data analysis

#### 2.6.1 Quality control

Raw data (raw reads) in FASTQ format were processed using in-house perl scripts. Clean data (clean reads) were obtained by removing reads containing adapter sequence, poly-Ns, and low-quality reads. At the same time, the Q20, Q30, and GC content of the clean data were calculated. All downstream analyses were performed using this high-quality clean data.

#### 2.6.2 Mapping to the reference genome

Reference genome and gene model annotation files were downloaded from the genome website directly. The index of the reference genome was built using Bowtie v2.06 and paired-end clean reads were aligned to the reference genome using TopHat v2.0.9 (see the Supplemental Content).

### 2.7 GO and KEGG enrichment analysis

GO enrichment analysis of differentially expressed genes or lncRNA target genes was implemented using the GOseq R package, with correction for gene length bias. GO terms with a corrected *P*-value <.05 were considered significantly enriched for differentially expressed genes.

KEGG is a database resource for understanding high-level functions and utilities of a biological system, at the cell, organism, and ecosystem levels, using molecular-level information, especially large-scale molecular datasets generated by genome sequencing and other high-throughput experimental technologies (http://www.genome.jp/kegg/). We used the KOBAS software to test for enrichment of differentially expressed genes or lncRNA target genes in KEGG pathways.

### 2.8 Validation of lncRNA expression in blood by RT-PCR

Total cellular RNA was isolated from peripheral blood samples using TRIzol reagent (Invitrogen, Carlsbad, CA), and cDNA was synthesized with the Takara PrimeScript RT Master Mix Kit (Takara Bio, Otsu, Japan). We used the ABI Prism 7500 sequence detection system (Applied Biosystems, Foster City, CA) to perform RT-PCR. lncRNAs were quantified using a quantitative real-time PCR (qRT-PCR) with KAPA SYBR Fast Universal (Kapa Biosystems Pty, South Africa). Briefly, reactions were performed in a mixture (20 μL) containing 1 μL cDNA template, 10 μL 2X SYBR-Green PCR Mix (kapa), 8 μL H_2_O, and 0.5 μL each of sense and antisense primers. A total of 7 lncRNAs were confirmed to be differentially expressed, using qRT-PCR. Table 2 shows the sequences of the primers used for RT-PCR. GADPH was used as the internal control. After qRT-PCR amplification, a melting curve analysis was performed to confirm reaction specificity, and the fold change (FC) of each lncRNA was calculated via the 2-ΔΔCt method.

**Table 2:**
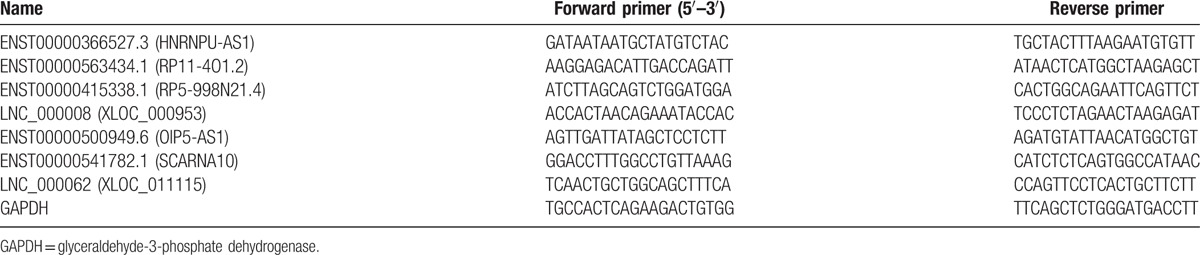
The listed primers were used to validate the expression of 7 lncRNAs.

### 2.9 Statistical analysis

SPSS v11.5 was used for all statistical analyses. Leukocyte cell counts are provided for individual samples in Table 1 for the purpose of eliminating cell count effects. Differentially expressed lncRNAs and mRNAs in peripheral blood samples were compared between EA and control samples, and a cut-off point of 2-fold for upregulation and 0.5-fold for downregulation of lncRNA expression were used. Student's *t*-test was used for gene expression analysis and a *P*-value ≤ .05 was considered statistically significant.

## 3 Results

**Figure 1:**
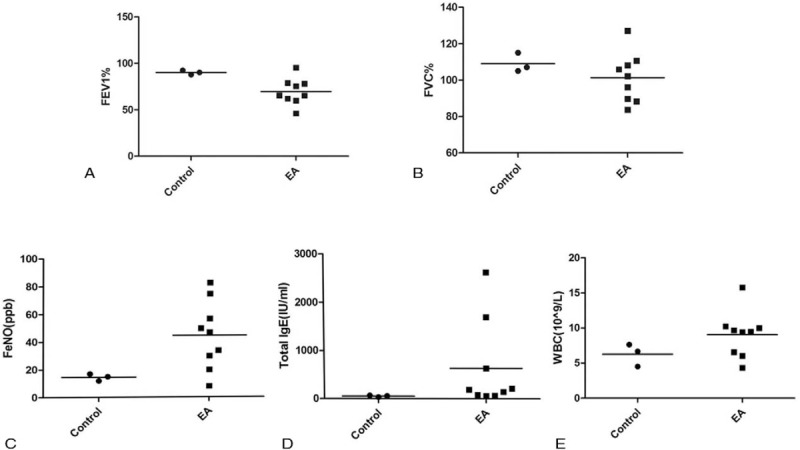
(A–E) The main clinical and laboratory features of the EA and control samples. EA = eosinophilic asthma.

The characteristics of the 9 EA samples (EAH n = 6 and EAL n = 3) and the 3 control samples are presented in Table 1 and Figure 1A–E. EA samples showed lower forced expiratory volume in the first second (FEV1)%, higher fractional exhaled nitric oxide (FeNO), and higher induced sputum eosinophil numbers. Most clinical variables did not differ between the EAH and EAL samples, but IgE in peripheral blood was significantly increased in EAH samples.

### 3.1 DGE analysis of mRNA and lncRNA

**Figure 2:**
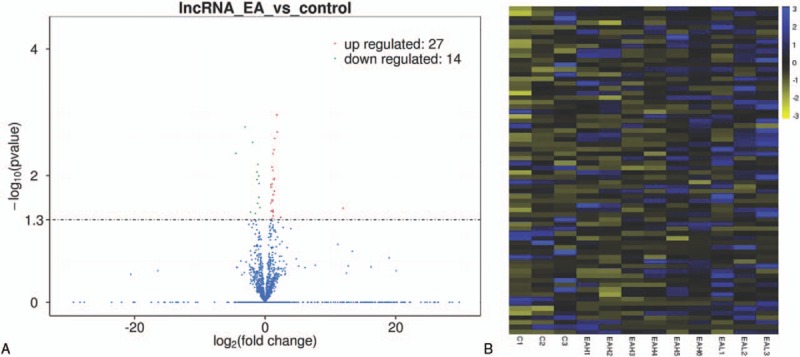
(A) Volcano plot assessment of lncRNA expression between EA and control groups. Red spots indicate a >2.0-fold change in lncRNA expression between EA and control groups. Green spots indicated a <0.5-fold change between EA and control groups. (B) Heat map analysis of differentially expressed lncRNAs between EA and control group. Yellow indicates low-lncRNA expression and blue indicates high-lncRNA expression. EA = eosinophilic asthma, lncRNA = long noncoding RNA.

**Figure 3:**
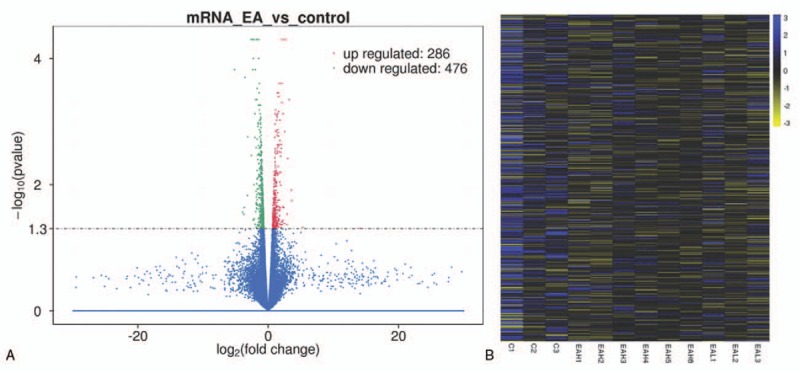
(A) Volcano plot assessment of mRNA expression between EA and control groups. Red spots indicate a >2.0-fold change in mRNA between EA and control groups. Green spots indicate a <0.5-fold change between EA and control groups. (B) Heat map analysis of differentially expressed mRNAs between EA and control group. Yellow indicates low-lncRNA expression and blue indicates high-lncRNA expression. EA = eosinophilic asthma, lncRNA = long noncoding RNA, mRNA = messenger RNA.

We analyzed the transcriptome of peripheral whole blood. The expression patterns differed significantly between EA and control samples. Using a 2-fold expression difference as a cutoff, a total of 41 lncRNA transcripts were specifically dysregulated (27 lncRNA transcripts upregulated and 14 lncRNAs transcripts downregulated; each *P* < .05) in EA compared with control samples (Fig. 2A and B). Additionally, a total of 762 mRNAs were specifically dysregulated including 286 mRNAs upregulated and 476 mRNAs downregulated in EA samples (Fig. 3A and B).

We also performed differential expression analysis for all pair-wise comparisons: EAH versus control samples, EAL versus control samples, and EAH versus EAL samples. In addition to the differences observed in gene expression between EA and control samples, EAH and EAL samples showed distinct gene expression profiles and clustered separately.

**Figure 4:**
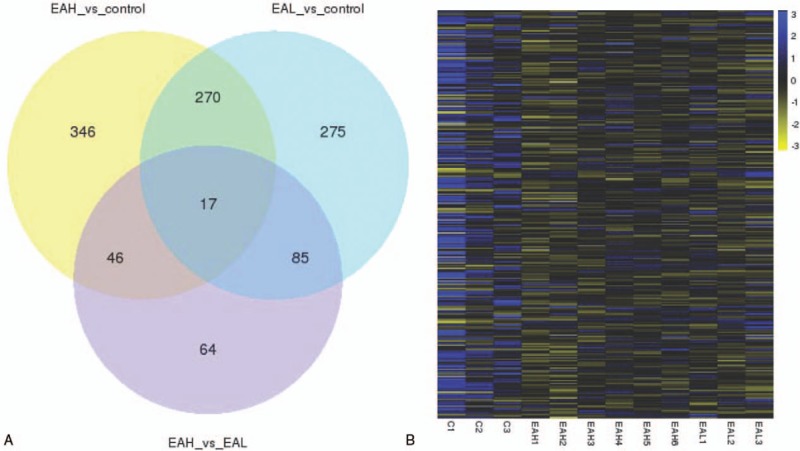
(A) Venn diagram showing differential expression of mRNAs between EA and control groups. (B) Heat map analysis of co-differentially expressed mRNAs, EAH vs control group plus EAL vs. control group (270 + 17 in [A]). Yellow indicates low-lncRNA expression and blue indicates high-lncRNA expression. EA = eosinophilic asthma, EAH = high-expression IgE group, EAL = low-expression IgE group, lncRNA = long noncoding RNA, mRNA = messenger RNA.

**Figure 5:**
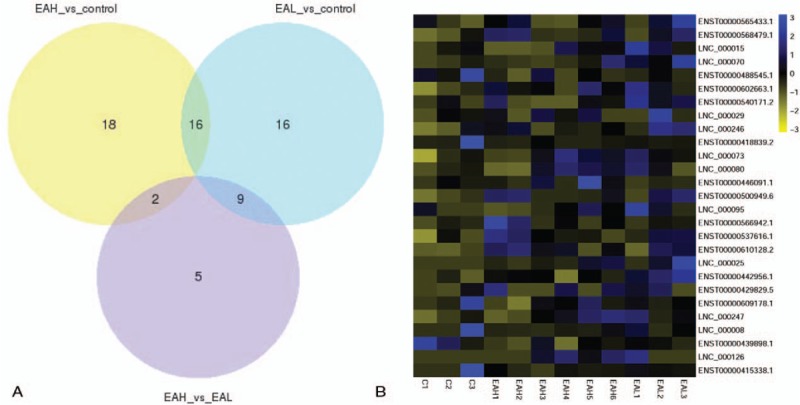
(A) Venn diagram showing differential expression of lncRNA between EA and control groups. (B) Heat map analysis of co-differentially expressed lncRNAs, EAH vs control, EAL vs control plus EAH vs EAL group (16 + 2 + 9 in [A]). Yellow indicates low-lncRNA expression and blue indicates high-lncRNA expression. EA = eosinophilic asthma, EAH = high-expression IgE group, EAL = low-expression IgE group, lncRNA = long noncoding RNA.

With respect to mRNA, a total of 1103 mRNAs were significantly differentially expressed (*P-*value ≤ .05). Venn diagrams and a heat map illustrating the overlap between the 2 differential expression analyses are shown in Figure 4A and B. The 2 sample sets showed distinct mRNA expression profiling pattern (see Tables E1–E4 Supplemental Content). Similarly, a total of 66 lncRNAs were differentially expressed (*P*-value ≤ .05). Venn diagrams and a heat map illustrating the overlap between the 2 differential expression analyses are shown in Figure 5A and B (see Tables E5–E8 Supplemental Content). The 2 sample sets showed distinct lncRNA expression profiling patterns.

**Figure 6:**
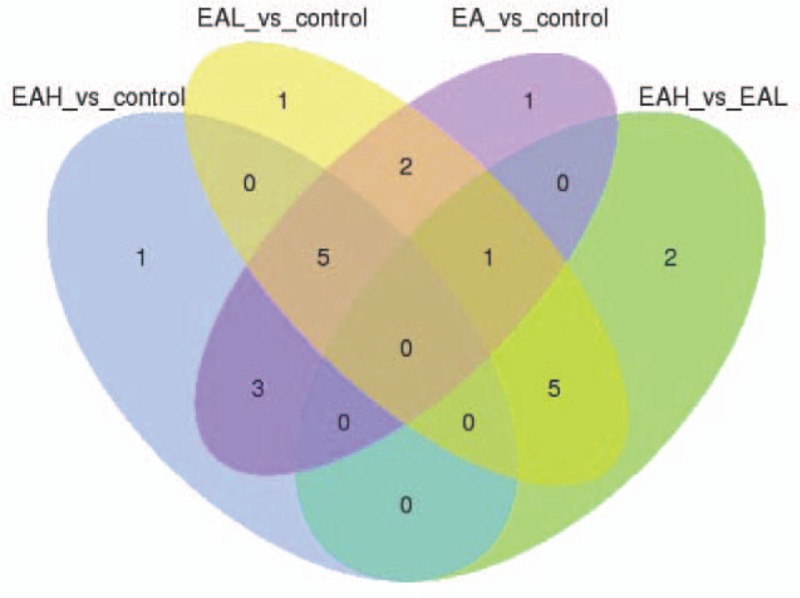
Venn diagram illustrating the overlap between the 2 different groups in expression of novel lncRNAs. lncRNA = long noncoding RNA.

Finally, 247 novel lncRNAs were identified, but most showed no differential expression between samples. Venn diagrams illustrating the overlap between the 2 differential expression analyses are shown in Figure 6.

### 3.2 Prediction of GO terms and KEGG pathway analysis for lncRNA between EA and control samples

**Figure 7:**
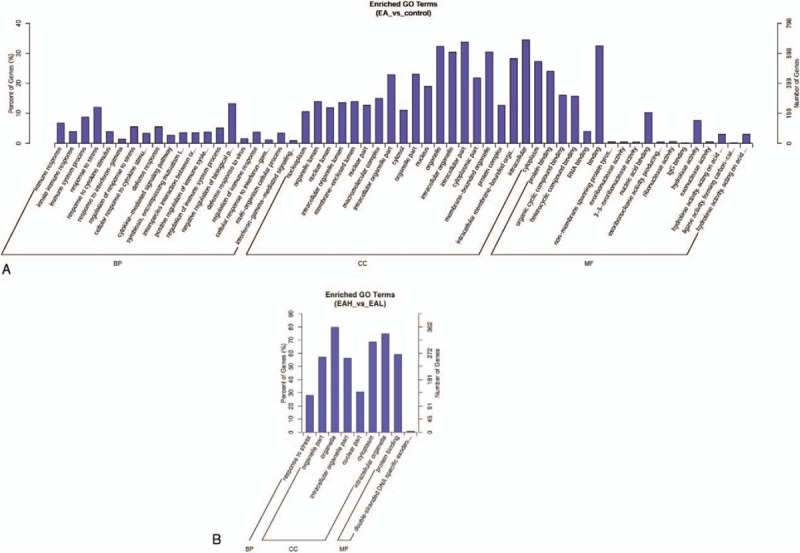
(A) Gene ontology (GO) analysis of differentially expressed lncRNAs between EA and control groups. The most enriched GO terms targeted by dysregulated transcripts were involved in a variety of functions, such as immune response, immune system process, response to stress, and negative regulation of biological process. (B) Gene ontology (GO) analysis of differentially expressed lncRNAs between EAH and EAL groups. The enriched GO term targeted by dysregulated transcripts was involved in response to stress. EAH = high-expression IgE group, EAL = low-expression IgE group, GO = gene ontology, lncRNA = long noncoding RNA.

**Figure 8:**
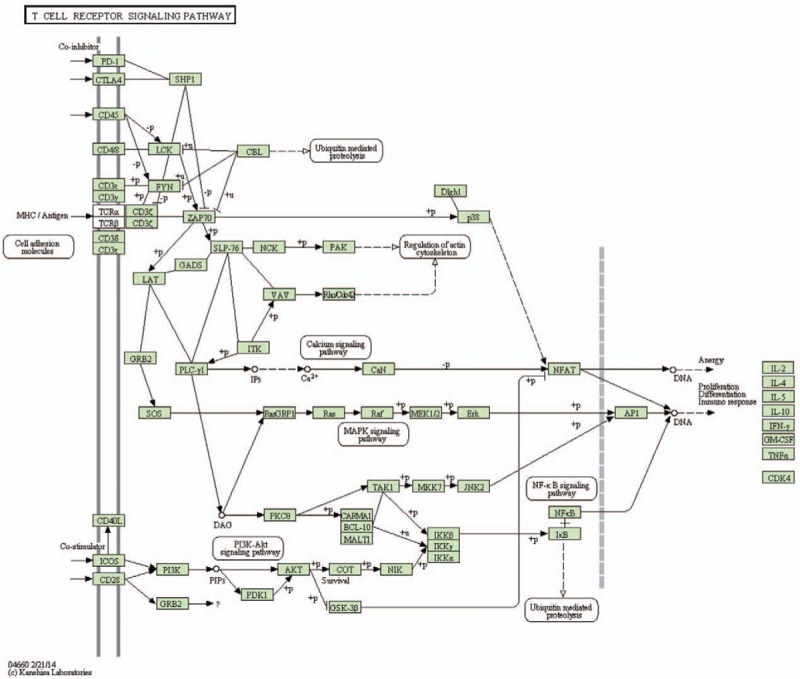
Kyoto Encyclopedia of Genes and Genomes (KEGG) pathway analysis of T cell receptor signaling pathways in EA. EA = eosinophilic asthma, KEGG = Kyoto Encyclopedia of Genes and Genomes.

We used *trans* and *cis* analysis to illustrate the correlation between lncRNA and mRNA expression. To explore the biology underlying the differentially expressed lncRNAs, we annotated them with gene symbols and searched for GO term enrichment using the GOseq R package, with correction for gene length bias. GO terms with a corrected *P*-value < .05 were considered significantly enriched. We used the KOBAS software to test for enrichment of differentially expressed genes or lncRNA target genes in KEGG pathways. Comparing EA with control samples using GO analysis showed that upregulated and downregulated transcripts were involved in immune response, immune system process, response to stress, and negative regulation of biological process (Fig. 7A). The most significant molecular function (MF) enrichment scores are also shown in Figure 7A. KEGG pathway annotation indicated that the most enriched pathways were Measles, T cell receptor signaling pathway, PPAR signaling pathway, Fc gamma R-mediated phagocytosis, NF-kappa, B signaling pathway, chemokine signaling pathway, and primary immunodeficiency (see Table E9 Supplemental Content). As one of the most important pathway, T cell receptor signaling pathway is listed in Figure 8.

### 3.3 GO term and KEGG pathway analysis of lncRNAs that differed between EAH and EAL samples

Comparing EAH with EAL samples using GO analysis showed that upregulated and downregulated transcripts were involved in response to stress (Fig. 7B). KEGG pathway annotation indicated that the most enriched pathways were apoptosis, toxoplasmosis, platelet activation, and dilated cardiomyopathy (see Table E10 Supplemental Content).

### 3.4 Prediction of lncRNA target genes associated with EA

We constructed a coding and noncoding gene co-expression network based on the correlation between differentially expressed lncRNAs and mRNAs. Pearson's correlation analysis was performed using a coefficient ≤ 0.95 to construct the network (see Table E11 Supplemental Content). We identified several genes associated with EA, including Il2RB, Il2RG, Il5RA, Il7R, Jak2, Stat2, Stat5A, TLSP, Ccl3, and Cxcl8, to identify co-expression lncRNAs. Among the 41 lncRNAs dysregulated between EA and control samples, 7 (HNRNPU-AS1, RP11-401.2, RP5-998N21.4, XLOC_000953, OIP5-AS1, SCARNA10, and XLOC_011115) were found to be co-expressed with these genes.

### 3.5 Confirmation of dysregulated lncRNAs in EA versus control samples

**Figure 9:**
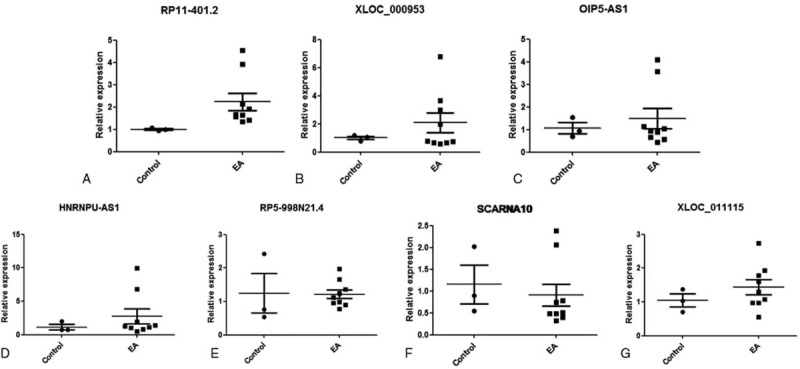
Seven selected lncRNA expression levels were validated in vivo. The expression of the selected seven lncRNAs was validated by qRT-PCR in 2 groups in vivo. lncRNA RP11-401.2 was upregulated in EA samples. lncRNA = long noncoding RNA.

To confirm the differentially expressed gene data, we further analyzed the above 7 dysregulated lncRNAs using qRT-PCR (Fig. 9). One lncRNA, RP11-401.2, was identified because its expression showed the highest conformance and stability, similar to those obtained from the sequencing analysis. RP11-401.2 showed significant differences in expression between the 2 groups in vivo; it was upregulated in the EA group. The results indicated that RP11-401.2 may be potential regulators of allergy.

## 4 Discussion

lncRNAs can be broadly divided into natural antisense, pseudogenes, long intergenic noncoding RNAs, and long intronic noncoding RNAs. lncRNAs are emerging as potential key regulators in gene expression networks and exhibit a surprising range of shapes and sizes.^[17,18]^ lncRNA was found to be involved in early differentiation of Th1 and Th2 by integrating transcriptional profiling data from multiple platforms.^[19]^ lncRNAs have been reported to exhibit distinct profiles in immune processes. A genome-wide RNA sequencing analysis showed that lncRNAs show differential expression in CD8 T cells;^[20]^ lncRNAs might be acting as enhancer elements during T-helper cell differentiation.^[21]^ The lncRNA BANCR is known to be upregulated in eosinophilic esophagitis, which is another allergic inflammatory disorder, and is induced in IL-13 in primary esophageal epithelial cells.^[22]^

To our knowledge, there is no report of lncRNA expression in human peripheral whole blood or its role in asthma, especially EA. In this study, we identified 41 lncRNAs and 271 mRNAs abnormally expressed in EA blood samples compared with control samples (fold change ≥2.0, *P* <.05). We found that some of these differentially expressed lncRNAs are involved in immune response, immune system process, and response to stress. These lncRNAs may regulate cell cycle progression and immune responses through various pathways, such as the T cell receptor signaling pathway, PPAR signaling pathway, Fc gamma R-mediated phagocytosis, NF-kappa B signaling pathway, chemokine signaling pathway, primary immunodeficiency, and the Jak-STAT signaling pathway. We demonstrated that RP11-401.2 was upregulated in EA samples using qRT-PCR. RP11-401.2 has been reported to be upregulated in TH2 cells,^[19]^ which are closely linked to EA. However, it remains to be determined how it participates in and contributes to EA progression or development.

A review summarized the role played by lncRNAs during T-lymphocyte development.^[23]^ TH1-specific lncRNA contains IFNG-AS1 and linc-MAF-4. IFNG-AS1 is induced in CD4+T cells in response to TH1 differentiation signals that require both stat4 and T-bet.^[24]^ Knockdown of linc-MAF-4 in activated CD4+T cells under nonpolarizing conditions decreases expression of TH1 lineage-specific mRNAs and increases expression of MAF, GATA3, IL4, and other TH2 lineage-specific mRNAs.^[25]^ TH2-specific lncRNAs include linc-Ccr2′5 AS, TH2LCRR, and GATA3-AS1. Depletion of linc-Ccr2′5 AS results in loss of Ccr1, Ccr2, Ccr3, and Ccr5.^[13]^ Depletion of TH2LCRR abrogates expression of IL4, IL5, and IL13 in human T cell cultures.^[26]^ GATA3-AS1 is present at high levels by CD4+ T cells.^[13]^ In our study, we also found that GATA3-AS1 differed significantly between EA and control samples.

Recent studies have reported that the lncRNA PVT1 is involved in asthma.^[27]^ PVT1 is decreased in patients with corticosteroid-sensitive nonsevere asthma and increased in patients with corticosteroid-insensitive severe asthma, and subsequent targeting studies demonstrated the importance of this lncRNA in controlling both proliferation and IL-6 release in ASMCs from patients with severe asthma.^[28]^ We identified PVT1 in our study, but noted no difference between samples. We attribute these differences to the fact that our lncRNAs were collected from peripheral whole blood rather than neutrophils, lymphocytes, monocytes, or adipose tissue.

In order to investigate whether a particular lncRNA influences IgE expression, we divided the EA samples into 2 groups (EAH and EAL). We found that mRNA expression of CD40LG expression was significantly increased in the EAH samples. In order for a B lymphocyte to switch to IgE production, it needs 2 signals provided by a Th2 cell in the form of the cytokines interleukin IL-4/IL-13 and CD40L.^[29,30]^ Through cis analysis, we found that lncRNA ENST00000454385.5 may play a role in IgE production.

In summary, because EA primarily affects the airways, it is useful to analyze gene expression in cells from the respiratory tract. Bronchoscopies, which are painful and invasive, are needed for the diagnosis of asthma, and it would be beneficial to develop noninvasive markers. Here we used peripheral whole blood, a sample which is easy to obtain and store and excellent for biomarker development. Our results provide a wealth of information on blood transcriptome lncRNA. Further study is now in progress to determine whether these lncRNAs may serve as new therapeutic targets and diagnostic markers for EA.

## Supplementary Material

Supplemental Digital Content

## Supplementary Material

Supplemental Digital Content

## Supplementary Material

Supplemental Digital Content

## Supplementary Material

Supplemental Digital Content

## Supplementary Material

Supplemental Digital Content

## Supplementary Material

Supplemental Digital Content

## Supplementary Material

Supplemental Digital Content

## Supplementary Material

Supplemental Digital Content

## Supplementary Material

Supplemental Digital Content

## Supplementary Material

Supplemental Digital Content

## Supplementary Material

Supplemental Digital Content

## Supplementary Material

Supplemental Digital Content
